# First Detection of Gammacoronavirus in a Striped Dolphin (*Stenella coeruleoalba*) from the Adriatic Sea

**DOI:** 10.3390/ani14182725

**Published:** 2024-09-20

**Authors:** Matteo Legnardi, Giovanni Franzo, Mattia Cecchinato, Haiyang Si, Riccardo Baston, Sandro Mazzariol, Cinzia Centelleghe, Guido Pietroluongo, Draško Holcer, Jure Miočić-Stošić, Jeroen Hofs, Maša Frleta-Valić, Claudia Maria Tucciarone

**Affiliations:** 1 Department of Animal Medicine, Production and Health (MAPS), University of Padua, Viale dell’Università 16, 35020 Legnaro, PD, Italy; matteo.legnardi@unipd.it (M.L.); giovanni.franzo@unipd.it (G.F.); mattia.cecchinato@unipd.it (M.C.); haiyang.si@phd.unipd.it (H.S.); riccardo.baston@phd.unipd.it (R.B.); 2 Department of Comparative Biomedicine and Food Science (BCA), University of Padua, Viale dell’Università 16, 35020 Legnaro, PD, Italy; sandro.mazzariol@unipd.it (S.M.); cinzia.centelleghe@unipd.it (C.C.); guido.pietroluongo@studenti.unipd.it (G.P.); 3 Croatian National History Museum, Demetrova 1, 10000 Zagreb, Croatia; drasko.holcer@hpm.hr; 4 Blue World Institute of Marine Research and Conservation, Kaštel 24, 51551 Veli Lošinj, Croatia; jure.miocicstosic@blue-world.org (J.M.-S.); jeroen.hofs@blue-world.org (J.H.); masa.frleta-valic@blue-world.org (M.F.-V.); 5 Marine Evolution and Conservation, Groningen Institute of Evolutionary Life Sciences, University of Groningen, P.O. Box 11103, 9747 AG Groningen, The Netherlands

**Keywords:** gammacoronavirus, cetacean, striped dolphin, Adriatic, morbillivirus

## Abstract

**Simple Summary:**

The present report describes the first molecular detection of a gammacoronavirus in a free-ranging striped dolphin coinfected with cetacean morbillivirus and found stranded on the Croatian coastline in 2022. The virus was detected in a heart sample and appeared different from previously identified cetacean gammacoronaviruses. This finding underscores the necessity of including this pathogen into routine diagnostics for stranded dolphins to gather important epidemiological data on coronavirus prevalence and its potential role in causing disease.

**Abstract:**

This case report presents the first molecular identification of a gammacoronavirus in a free-ranging striped dolphin (*Stenella coeruleoalba*) that was found stranded along the Croatian coastline in 2022. The dolphin exhibited a concurrent infection with cetacean morbillivirus. The gammacoronavirus strain was amplified and sequenced from heart tissue imprinted on an FTA^®^card, revealing a notable genetic distance (approximately 8%) from previously characterized cetacean gammacoronaviruses. This finding highlights the importance of including gammacoronaviruses in routine diagnostics for stranded dolphins to gather epidemiological data on their prevalence and potential role in causing disease in cetaceans. This study sets the premises for a further understanding of the diversity and distribution of gammacoronaviruses in marine mammals and highlights the necessity for ongoing surveillance of emerging infectious diseases in wild populations.

## 1. Introduction

Marine mammals are looked at with increasing interest and attention, due to their apical position in the trophic web and their role as indicators of marine environmental health. However, one-quarter of cetacean species is threatened by extinction [1], in an escalating trend over the past thirty years. Natural factors such as habitat characteristics and prey distribution influence cetacean populations, but anthropogenic factors are becoming predominant in altering the global ecology [2].

In the context of emerging pathogens, climate change, anthropization, and food and habitat resources create favorable conditions for the emergence, spread, or spillover of new threats to both human and animal populations. Habitat changes and the unprecedented overlap of species also lead to new contacts and transmission routes, increasing disease susceptibility and pathogen circulation [3]. The rise in pathogen detection, including some with zoonotic potential, is notable among stranded animals, with frequent detections of *Brucella ceti*, *Toxoplasma gondii*, and influenza A virus [3,4].

A contribution to an impaired immune status can be given by primary agents, such as *Cetacean morbillivirus* (CeMV), which is a highly contagious, single-stranded RNA virus commonly affecting various species of cetaceans. CeMV can cause severe respiratory and neurological signs and promote secondary infections due to immunosuppression [5]. Along with CeMV and often in coinfection, herpesviruses are frequently detected, and two subfamilies can infect cetaceans (*Alphaherpesvirinae* and *Gammaherpesvirinae*) [6].

Coronaviruses and their host tropisms have garnered interest, particularly post-Severe Acute Respiratory Syndrome Coronavirus 2 (SARS-CoV-2) pandemic. Although a certain similarity of the SARS-CoV-2 receptor (angiotensin-converting enzyme-2, ACE-2) in cetaceans and humans could constitute a predisposing factor for infection [7], SARS-CoV-2 has not been detected in cetaceans yet. Surprisingly, coronaviruses belonging to the *Gammacoronavirus* genus have been identified in cetaceans, but only in captive animals so far: in liver samples of a beluga whale (*Delphinapterus leucas*) that died in an aquatic park in the US [8], in fecal samples from symptomatic common bottlenose dolphins (*Tursiops truncatus*) in the US [9], and from Indo-Pacific bottlenose dolphins (*Tursiops aduncus*) in Hong Kong (Acc. Num. KF793824-KF793826).

Even if several countries have started monitoring activities on stranded animals, epidemiological information on coronaviruses in wild marine mammals is lacking. The systematic diagnostic approach should include the research of various pathogens, especially less common agents, whose niche could expand to different geographic areas and species. Such a statement is enforced by the present report of a coronavirus detection in a free-ranging striped dolphin stranded along the Adriatic coastline.

## 2. Materials and Methods

A striped dolphin (*Stenella coeruleoalba*) was found stranded alive on Milna Bay beach, Vis Island, Croatia, on 24 April 2022. After unsuccessful refloating attempts, the animal died and was immediately frozen and transported to the Croatian Veterinary Institute in Rijeka for necropsy. Necropsy was performed by trained pathologists of the Comparative Biomedicine and Food Science (BCA) Department (CITES n°IT020), University of Padua, and staff of the Blue World Institute within capacity-building activities of the LIFE DELFI project (LIFE18 NAT/IT/000942), following standard protocols.

Tissue samples were collected for virological analysis from target organs (brain, lung, prescapular and pulmonary lymph nodes, heart, liver, and kidney) and imprinted on FTA^®^cards using standardized procedures [10] and were sent to the Animal Medicine Production and Health (MAPS) Department, University of Padua, for virological examinations.

Nucleic acids were extracted from FTA^®^cards as previously described [10] and tested for internal control detection [11]. Samples were tested for dolphin morbillivirus (DMV) with a nested RT-PCR assay targeting the H gene [12] and for coronavirus using an RT-PCR assay targeting the RdRp gene, with a forward primer (5′-GGTTGGGACTATCCTAAGTGTGA-3′) from a published method [13,14] and a reverse primer (5′-CACAACACCATCATCGCTCA-3′) specific for bottlenose dolphin coronavirus (MN690611.1) and beluga whale coronavirus (EU111742.1). The assay was validated in house on serial dilutions of a plasmid containing a 447 bp-long sequence from MN690611, showing a limit of detection (LoD) of 10^1^ copies/µL. Specificity was assessed on other coronaviruses (infectious bronchitis virus IBV, bovine coronavirus BCoV), and the final thermal protocol was the following: 30 min at 50 °C for retrotranscription, 2 min at 94 °C for activation, 45 cycles of 2 min at 94 °C for denaturation, 20 s at 53 °C for annealing, and 40 s at 68 °C for extension, with a 5-min long final extension phase at 68 °C.

Both assays were performed using the SuperScript III One-Step RT-PCR System with the Platinum Taq DNA Polymerase kit, and the Platinum™ II Taq Hot-Start DNA Polymerase kit (Thermofisher Scientific, Waltham, MA, USA) was used for the DMV nested PCR. Herpesvirus presence was investigated with a previously validated pan-herpesvirus nested PCR [15]. Both amplification rounds were performed with the abovementioned PCR kit, and all assays were run on an Applied Biosystems 2720 Thermal Cycler (Thermofisher Scientific, USA). A DMV viral isolate, a feline herpesvirus vaccine aliquot, and a dilution of the plasmid used for the coronavirus assay validation were used as positive controls. 

PCR products were Sanger-sequenced in forward and reverse directions using the amplification primer pair at Macrogen Europe (Milan, Italy). A preliminary evaluation of the sequences was performed via BLAST search, then Maximum Likelihood phylogenetic trees were reconstructed using MEGA X software [16], along with reference sequence datasets (Table 1 and Table 2), to characterize the strain relationships when possible. 

## 3. Results and Discussion

The animal was a 170 cm-long adult female with a moderate decomposition code of the carcass (DCC3) [17]. During external examination, poor nutritional condition and external wounds, likely related to the stranding event, were noted, along with multifocal rare small parasitic arthropods compatible with *Pennella* spp. At gross examination, generalized congestion of the gastrointestinal tract was evident, along with mild to moderate parasitic presence (*Anisakis* spp. in the first concameration, nodules of *Pholeter gastrophilus* in the second and third ones). A focal abscess was observed affecting the mammary glands, while the lungs appeared inflated with an evident rib mark, and a generalized reactive lymphadenopathy was noticed, with marked enlargement in prescapular and pulmonary lymph nodes. In most of the tissues, morphological changes were not detected also due to freezing and defrosting processes, which impaired microbiological examinations. Despite freezing artifacts, a multifocal chronic meningoencephalitis was noted, while no relevant pathological findings were visible in the most preserved tissues, such as the heart and lungs.

Regarding molecular analyses, internal control was detected on all extracted samples (RNA Cq: 21.40–28.60, median 26.93, mean 25.53; DNA Cq: 17.84–21.82, median 21.43, mean 20.95), confirming extraction. All samples tested negative for herpesvirus. DMV was identified only in the sample obtained from the prescapular lymph node, while the heart sample was positive for coronavirus.

**Figure 1:**
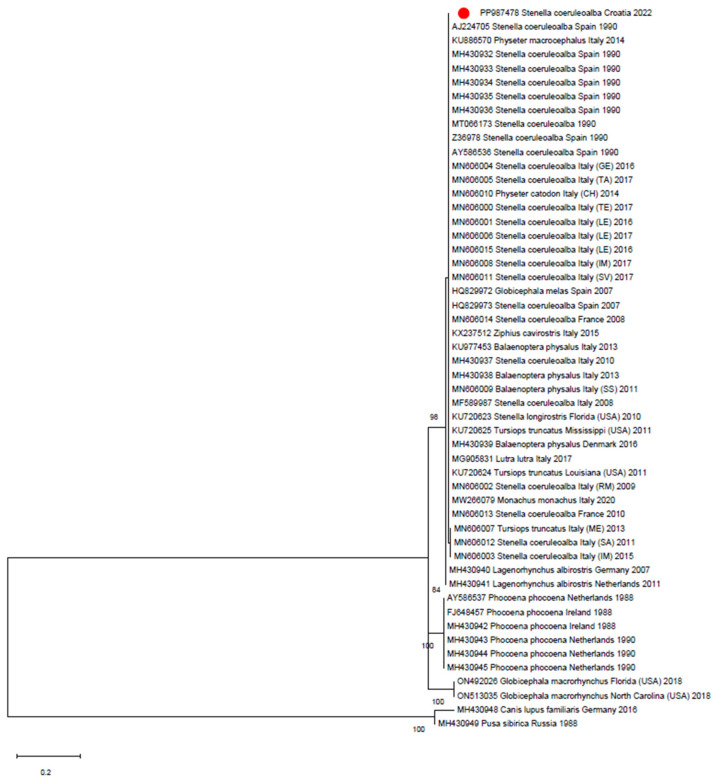
Maximum likelihood phylogenetic tree reconstructed on the partial H gene of CeMV sequences, including dolphin morbillivirus, pilot whale morbillivirus, porpoise morbillivirus, and distemper virus reference strains. The red dot indicates the Croatian sequence. The tree was reconstructed using the maximum likelihood method and Tamura-3-parameter substitution model with invariable sites. Bootstrap support (>70%) is shown next to the branches. This analysis involved 52 nucleotide sequences. The final dataset was composed of 158 positions.

A 158-bp sequence was obtained from the DMV-positive sample, confirming the amplicon specificity (Acc. Num. PP987478). However, the short sequence length limited further strain classification (Figure 1). While typical microscopic brain lesions consistent with DMV-related pathology were observed, the limited sampling and the artifacts affecting the examined tissues did not allow to ascertain the role of the morbillivirus infection as the possible cause of death.

**Figure 2:**
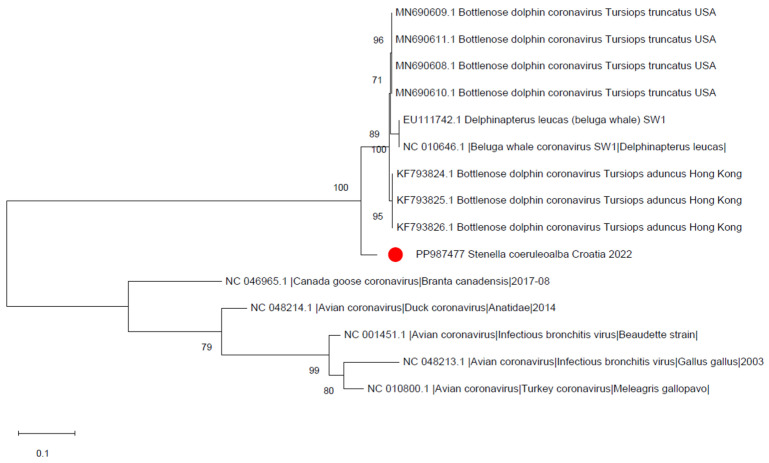
Maximum likelihood phylogenetic tree reconstructed on the partial RdRp gene of gammacoronavirus sequences using the Tamura 3-parameter substitution model with Gamma distribution (G). Bootstrap support (>70%) is shown next to the branches. This analysis involved 15 nucleotide sequences. The final dataset was composed of 392 positions. The red dot indicates the Croatian sequence.

A 392-bp sequence was obtained from the coronavirus amplicon (Acc. Num. PP987477), showing a percentage of identity of 92.21% with beluga whale coronavirus SW1 (Acc. Num. EU111742) and 93.26% with bottlenose dolphin coronavirus (Acc. Num. MN690611). The differences between the retrieved sequence and the lab’s positive control ruled out contamination, and the sequence relationship with reference sequences is represented in Figure 2.

Whole genome sequencing of the detected gammacoronavirus was attempted by NGS. A library was prepared from total RNA with the SMARTer stranded low RNA (Ribo-Zero) kit, and sequencing was performed on an Illumina platform at Macrogen Europe (Milan, Italy). However, the library quality check failed, likely due to nucleic acid fragmentation on the FTA^®^cards.

The obtained short sequence is insufficient for a comprehensive characterization of the coronavirus strain. Nonetheless, the used RT-PCR assay targets a generally well-conserved region of the RNA-dependent RNA polymerase (RdRp) gene. Therefore, the observed diversity compared to previously sequenced strains from cetacean hosts is noteworthy. A marked heterogeneity was previously observed among cetacean coronavirus strains, which differ substantially at the spike gene level, the most variable region. The distance in the RdRp gene herein detected suggests a much greater divergence at the full genome level, so further analyses are required to better characterize this virus.

Many gaps hamper the reconstruction of the evolutionary and spreading patterns of gammacoronaviruses in cetaceans, highlighting the need to include these pathogens in routine diagnostic panels. This would aid in understanding their tissue tropism, pathogenesis, and epidemiology. In fact, the lack of lesions commonly imputable to coronaviruses, in particular in the heart tissue, hampers any speculation on pathogenicity based on the presently described case, which is not informative due to the poor yield of histology. So far, cetacean coronaviruses have been detected in liver [8], fecal [9], and heart samples (this study) of different species from different areas, providing limited insights into pathogenesis. Viral presence in feces could indicate a classical enteric tropism or shedding route following systemic infection, whereas detection in liver and heart might imply systemic infection or specific tropism. The detection of similar viruses in animals from such distant and segregated habitats might indicate a well-established presence of gammacoronaviruses in aquatic populations and separate evolution from a common ancestor, partially explaining the genetic distance herein described. Further studies and cases will be necessary to start collecting data on coronavirus pathogenesis and its role in cetaceans, as well as the possible interactions with other pathogens, such as morbillivirus, which could have caused immunosuppression, possibly favoring a secondary infection sustained by the coronavirus. 

## 4. Conclusions

Remarkably, to the best of our knowledge, this is the first report of coronavirus detection in a free-ranging cetacean. The poor genetic yield of the samples could have been influenced by the state of the carcass at the time of necropsy and sampling. These constraints, coupled with the lack of systematic coronavirus screening, may contribute to the underestimation of the actual infection frequency in cetaceans, that should be further investigated to broaden the knowledge of coronavirus genetic diversity, host range, epidemiology, and pathogenic features.

## Figures and Tables

**Table 1:** List of accession numbers of CeMV reference sequences and relative metadata.

Accession Number	Host Species	Country	Collection Date
**PP987478**	***Stenella coeruleoalba***	**Croatia**	**2022**
AJ224705	*Stenella coeruleoalba*	Spain	1990
AY586536	*Stenella coeruleoalba*	Spain	1990
AY586537	*Phocoena phocoena*	Netherlands	1988
FJ648457	*Phocoena phocoena*	Ireland	1988
HQ829972	*Globicephala melas*	Spain	2007
HQ829973	*Stenella coeruleoalba*	Spain	2007
KU720623	*Stenella longirostris*	Florida (USA)	2010
KU720624	*Tursiops truncatus*	Louisiana (USA)	2011
KU720625	*Tursiops truncatus*	Mississippi (USA)	2011
KU886570	*Physeter macrocephalus*	Italy	2014
KU977453	*Balaenoptera physalus*	Italy	2013
KX237512	*Ziphius cavirostris*	Italy	2015
MF589987	*Stenella coeruleoalba*	Italy	2008
MG905831	*Lutra lutra*	Italy	2017
MH430932	*Stenella coeruleoalba*	Spain	1990
MH430933	*Stenella coeruleoalba*	Spain	1990
MH430934	*Stenella coeruleoalba*	Spain	1990
MH430935	*Stenella coeruleoalba*	Spain	1990
MH430936	*Stenella coeruleoalba*	Spain	1990
MH430937	*Stenella coeruleoalba*	Italy	2010
MH430938	*Balaenoptera physalus*	Italy	2013
MH430939	*Balaenoptera physalus*	Denmark	2016
MH430940	*Lagenorhynchus albirostris*	Germany	2007
MH430941	*Lagenorhynchus albirostris*	Netherlands	2011
MH430942	*Phocoena phocoena*	Ireland	1988
MH430943	*Phocoena phocoena*	Netherlands	1990
MH430944	*Phocoena phocoena*	Netherlands	1990
MH430945	*Phocoena phocoena*	Netherlands	1990
MH430948	*Canis lupus familiaris*	Germany	2016
MH430949	*Pusa sibirica*	Russia	1988
MN606000	*Stenella coeruleoalba*	Italy (TE)	2017
MN606001	*Stenella coeruleoalba*	Italy (LE)	2016
MN606002	*Stenella coeruleoalba*	Italy (RM)	2009
MN606003	*Stenella coeruleoalba*	Italy (IM)	2015
MN606004	*Stenella coeruleoalba*	Italy (GE)	2016
MN606005	*Stenella coeruleoalba*	Italy (TA)	2017
MN606006	*Stenella coeruleoalba*	Italy (LE)	2017
MN606007	*Tursiops truncatus*	Italy (ME)	2013
MN606008	*Stenella coeruleoalba*	Italy (IM)	2017
MN606009	*Balaenoptera physalus*	Italy (SS)	2011
MN606010	*Physeter catodon*	Italy (CH)	2014
MN606011	*Stenella coeruleoalba*	Italy (SV)	2017
MN606012	*Stenella coeruleoalba*	Italy (SA)	2011
MN606013	*Stenella coeruleoalba*	France	2010
MN606014	*Stenella coeruleoalba*	France	2008
MN606015	*Stenella coeruleoalba*	Italy (LE)	2016
MT066173	*Stenella coeruleoalba*		1990
MW266079	*Monachus monachus*	Italy	2020
ON492026	*Globicephala macrorhynchus*	Florida (USA)	2018
ON513035	*Globicephala macrorhynchus*	North Carolina (USA)	2018
Z36978	*Stenella coeruleoalba*	Spain	1990

**Table 2:** List of accession numbers of gammacoronavirus reference sequences and relative metadata.

Accession Number	Host Species	Genus/Species	Strain	Collection Date
PP987477	***Stenella coeruleoalba***		ID586	**2022**
EU111742.1	*Delphinapterus leucas*	*Cegacovirus*	SW1	
NC_010646.1	*Delphinapterus leucas*	*Cegacovirus*	SW1	
KF793824.1	*Tursiops aduncus*	*Cegacovirus*	Bottlenose dolphin coronavirus HKU22 isolate CF090325	
KF793825.1	*Tursiops aduncus*	*Cegacovirus*	Bottlenose dolphin coronavirus HKU22 isolate CF090327	
KF793826.1	*Tursiops aduncus*	*Cegacovirus*	Bottlenose dolphin coronavirus HKU22 isolate CF090331	
MN690608.1	*Tursiops truncatus*	*Cegacovirus*	Bottlenose dolphin coronavirus strain 37112-1	
MN690609.1	*Tursiops truncatus*	*Cegacovirus*	Bottlenose dolphin coronavirus strain 37112-2	
MN690610.1	*Tursiops truncatus*	*Cegacovirus*	Bottlenose dolphin coronavirus strain 37112-3	
MN690611.1	*Tursiops truncatus*	*Cegacovirus*	Bottlenose dolphin coronavirus strain 37112-4	
NC_001451.1	*Gallus gallus*	*Igacovirus/Avian coronavirus*	Infectious bronchitis virus	
NC_010800.1	*Meleagris gallopavo*	*Igacovirus/Avian coronavirus*	Turkey coronavirus	
NC_048213.1	*Gallus gallus*	*Igacovirus/Avian coronavirus*	Infectious bronchitis virus	2003
NC_048214.1	*Anatidae*	*Igacovirus/Avian coronavirus*	Duck coronavirus	2014
NC_046965.1	*Branta canadensis*	*Brangacovirus/Goose coronavirus*	Goose coronavirus CB17	2017

## Data Availability

The original contributions presented in the study are included in the article, further inquiries can be directed to the authors.
